# LINKING DEMENTIA CAREGIVING EXPERIENCES WITH EXPECTATIONS OF FUTURE HEALTH CARE NAVIGATION

**DOI:** 10.1093/geroni/igae098.0864

**Published:** 2024-12-31

**Authors:** Rachel Bloom, Yifan Lou, Joan Monin, Terri Fried, Francesca Falzarano, Emily Mroz

**Affiliations:** Fordham University, New York, New York, United States; Virginia Commonwealth University, New Haven, Virginia, United States; Yale School of Public Health, New Haven, Connecticut, United States; Yale University School of Medicine, New Haven, Connecticut, United States; University of Southern California, Los Angeles, California, United States; Emory University, Decatur, Georgia, United States

## Abstract

Caregivers of people living with advanced dementia are often exposed to the complexities of healthcare coordination and decision-making over the disease trajectory. These observations can shape former caregivers’ expectations of healthcare available to them in the future, particularly approaching their end-of-life. We conducted a reflexive thematic analysis to characterize adult-child caregivers’ observations about healthcare provision for people with dementia, as well as exploring how and whether these former caregivers related these observations to their own end-of-life care expectations. Analysis of semi-structured interviews from 32 bereaved caregivers generated two themes describing how caregiving influenced caregivers’ understanding of a) the need for medical advocacy and b) the appropriateness of medical services at the end-of-life. For some former caregivers, having engaged in medical advocacy for their parents served to demystify the process of communicating with medical professionals about goals of care and the relative utility of medical interventions, fostering feelings of preparedness for self-advocacy around receiving what they judge to be appropriate care in the future. Some participants linked their observations to changes in their perceptions of hospice care, with the majority of this group expressing an increased appreciation for its role in enhancing comfort and reducing care fragmentation. Others indicated that caregiving reinforced their existing appraisal of hospice as appropriate for improving end-of-life experiences. This work indicates that former dementia caregivers’ healthcare observations shape their broader understanding of end-of-life opportunities and limitations within current medical systems, and influence their perceived ability to navigate similar contexts in the future.

